# Inhibition of ISAV Membrane Fusion by a Peptide Derived from Its Fusion Protein

**DOI:** 10.3390/membranes15060180

**Published:** 2025-06-15

**Authors:** María Elena Tarnok, Lucía Caravia-Merlo, Constanza Cárdenas, Fanny Guzmán, Luis F. Aguilar

**Affiliations:** 1 Instituto de Química, Pontificia Universidad Católica de Valparaíso, Valparaíso 2373223, Chile; maria.tarnok@pucv.cl (M.E.T.); lucia.caravia.m@mail.pucv.cl (L.C.-M.); 2 Núcleo de Biotecnología Curauma (NBC), Pontificia Universidad Católica de Valparaíso, Valparaíso 2373223, Chile; constanza.cardenas@pucv.cl (C.C.); fanny.guzman@pucv.cl (F.G.)

**Keywords:** fusion peptide, membrane fusion, viral fusion

## Abstract

Peptides designed to interfere with specific steps of viral infection mechanisms have shown promising antiviral potential. In this study, we investigated the ability of a synthetic peptide (peptide 303), derived from the fusion protein sequence of the Infectious Salmon Anemia Virus (ISAV), to inhibit membrane fusion mediated by the ISAV fusion peptide (ISAV-FP1). To assess this, we employed a model membrane system consisting of large unilamellar vesicles (LUVs) composed of 1,2-dioleoyl-sn-glycero-3-phosphocholine (DOPC), dipalmitoylphosphatidylcholine (DPPC), and cholesterol. Membrane fusion kinetics were monitored via R18 fluorescence dequenching. Additionally, the interaction of peptide 303 with lipid membranes was evaluated using fluorescence anisotropy measurements. The potential direct interaction between peptide 303 and ISAV-FP1 was further examined through Förster Resonance Energy Transfer (FRET) assays. Our results demonstrate that peptide 303 effectively inhibits ISAV-FP1-mediated membrane fusion. Furthermore, peptide 303 was shown to interact with lipid bilayers and with ISAV-FP1 itself. These findings suggest a dual inhibitory mechanism in which peptide 303 both prevents ISAV-FP1 binding to the membrane and directly interacts with the fusion peptide, thereby disrupting its fusogenic activity.

## 1. Introduction

The Infectious Salmon Anemia Virus (ISAV) has had severe economic impacts on the global salmon aquaculture industry. This pathogen is the causative agent of Infectious Salmon Anemia (ISA), a highly virulent disease primarily affecting Atlantic salmon (*Salmo salar*), one of the most commercially important fish species [1]. ISAV is an enveloped virus with a segmented, negative-sense single-stranded RNA genome, surrounded by a lipid membrane. It belongs to the *Isavirus* genus within the *Orthomyxoviridae* family [2]. This virus has been reported in all major salmon-producing countries, including Norway, Canada, and Chile [3,4,5]. In Chile, a significant outbreak in 2007 led to a 64% reduction in salmon production [6]. ISAV is transmitted horizontally through water and infected hosts, and its spread is exacerbated by ectoparasites, such as *Caligus rogercresseyi*, which can act as mechanical vectors when co-infected [7]. The growing global demand for salmon has driven the industry toward higher stocking densities, increasing physiological stress in fish and thereby elevating susceptibility to infections and disease outbreaks [8].

A critical step in the infection mechanism of enveloped viruses, including the ISAV, is the fusion of the viral envelope with the host cell membrane. This membrane fusion is mediated by specialized viral fusion proteins embedded in the lipid envelope. Aspehaug et al. [9] characterized the fusion protein (F protein) of ISAV as a 50 kDa glycoprotein. In the ISAV infection mechanism, two proteins are key to the association and fusion of the viral membrane with the host membrane, with these being hemagglutinin esterase (HE) and the F protein. The functional domain of HE allows binding to the 4-O-acetylated sialic acid receptor on the surface of the host membrane, while the acetylesterase activity destroys the sialic acid bonds, promoting virus release [10]. The F protein is responsible for fusion with the host membrane, facilitating the entry of viral genetic material for replication [11]. This protein is found as a precursor in the viral membrane, called F0. It is cleaved by a proteolytic protein of the host cell for activation; this cleavage results in two subunits: F1 (20 kDa) and F2 (30 kDa), linked by disulfide bridges [12]. The amino-terminal region of the F2 subunit, generated by proteolytic cleavage of the fusion precursor, contains a sequence known as the fusion peptide. In a previous study, we demonstrated that the ISAV fusion mechanism also depends on the lipid composition of target membranes, particularly influenced by the cholesterol content [13]. Consequently, one potential strategy for developing novel inhibitors of ISAV infection involves identifying molecules capable of disrupting the lipid ordering induced by cholesterol, thereby hindering membrane fusion.

Currently, there are no molecular strategies available for the prevention or treatment of ISA. One promising approach involves the use of peptides to block viral infection. This strategy has been explored in the context of other viruses, such as the influenza virus, where several antiviral peptides have been identified that can interfere with different stages of the viral life cycle. For example, Rajik et al. investigated antiviral peptides against the H9N2 avian influenza virus, selecting candidates from a phage display library. They identified a novel peptide capable of binding to viral hemagglutinin [14].

**Figure 1:**
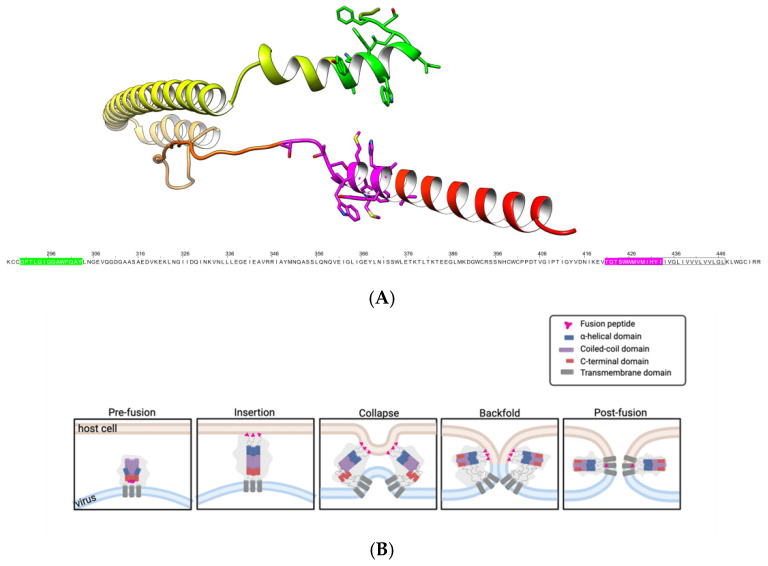
Structural model of the F2 subunit of the ISAV fusion protein and schematic representation of the membrane fusion mechanism mediated by class I fusion proteins. (**A**) C-terminal region of the ISAV fusion protein (residues 276–444). Peptide ISAV-FP1 is highlighted in green, and peptide 303 is shown in magenta. The transmembrane domain is indicated by a black box in the reference sequence and represented as a flat helix in the structural model. (**B**) General model of the membrane fusion mechanism for class I fusion proteins. The fusion peptide (ISAV-FP1) and peptide 303, located within the transmembrane domain, are indicated. The diagram illustrates the sequential stages of membrane fusion: pre-fusion, insertion, collapse, backfold, and post-fusion.

Studies on viruses with class I fusion proteins, such as ISAV and human influenza virus, suggest that the trimeric fusion protein undergoes pH-dependent conformational changes within endosomes, exposing the fusion peptide, which inserts into the host membrane and facilitates fusion between the viral and cellular membranes, as illustrated in Figure 1 [12,15,16,17]. To provide a more detailed description, membrane fusion mediated by class I viral fusion proteins follows a series of conformational transitions that ultimately drive the fusion of the viral and host membranes (as shown in Figure 1B). In their native pre-fusion conformation, these trimeric proteins remain metastable until activated by environmental cues, such as an acidic pH within endosomes, as is the case for ISAV. Upon activation, the protein undergoes a structural rearrangement that exposes a hydrophobic fusion peptide, which inserts into the host membrane; this marks the insertion stage. Subsequently, the protein collapses into a hairpin-like structure, a step known as collapse, drawing the viral and host membranes into close proximity. This is followed by the backfolding phase, in which the heptad repeat regions of the fusion protein refold into a six-helix bundle, effectively pulling the membranes together. These intermediate states facilitate hemifusion, where the outer leaflets of the lipid bilayers merge. In this step the fusion peptide approaches the transmembrane segments of the fusion protein [11,15,16]. Finally, the formation of a post-fusion structure leads to the opening of a fusion pore, completing the fusion process.

Considering these membrane interactions between segments of different fusion protein sequences, in our research we evaluated if a peptide designed from the transmembrane domain of the ISAV fusion protein (peptide 303) was able to interact with an ISAV fusion peptide and inhibit ISAV-facilitated membrane fusion. The fusion protein of ISAV (UniProt ID: B1NWL9) was used to generate a structural model using the AlphaFold server [18]. The location of the peptides is shown alongside the reference sequence in Figure 1A. Figure 1A shows the proximity of the peptide 303 segment to the fusion peptide segment when the hairpin structure of the protein is formed in the collapse step.

## 2. Materials and Methods

### 2.1. Reagents

Cholesterol (CHO), 1,2-dioleoyl-sn-glycero-3-phosphocholine (DOPC) and dipalmitoylphosphatidylcholine (DPPC) were obtained from Avanti Polar Lipids (Alabaster, AL, USA); 1,6-diphenyl-1,3,5-hexatriene (DPH), 1-(4-trimethylammonium)-6-phenyl-1,3,5-hexatriene (TMA-DPH), 6-lauroyl-2-dimethylaminonaphthalene (Laurdan), and octadecyl rhodamine B chloride (R18) were obtained from Invitrogen (Thermo Fisher Scientific, Waltham, MA, USA). Triton X-100 (TX-100) was purchased from Sigma Aldrich (St. Louis, MO, USA).

### 2.2. Peptide Synthesis and Characterization

GFTLGIGGAWFQAY (ISAV-FP1) and TGTSWWMVMIHYI (peptide 303) peptides were synthesized by solid-phase peptide synthesis (SPPS) using the Fmoc/tBu strategy, as previously described by Guzmán et al [19]. Following synthesis, the peptides were purified using C18 reversed-phase chromatography and characterized by analytical high-performance liquid chromatography (HPLC) and mass spectrometry.

The incorporation of fluorophores was performed using the same coupling procedure as for amino acids during the synthesis cycle. Fluorescein was attached to peptide ISAV-FP and rhodamine to peptide 303.

### 2.3. Preparation of Vesicles

Large unilamellar vesicles (LUVs) were prepared with a lipid mixture of DOPC/DPPC in a molar ratio of 1:1 with 20% molar cholesterol relative to total lipids. A corresponding amount of the lipid mixture dissolved in chloroform was added to a glass bottle and the chloroform was evaporated under N_2_ flow until a dry lipid layer was obtained and the lipid mixture was resuspended in a 20 mM citrate buffer at pH 4.5. Then, this solution was heated to 60 °C and homogenized by vortexing. Subsequently, 10 freeze–thaw cycles were performed using liquid nitrogen and a thermoregulated bath at 60 °C. Finally, the liposome solution was passed 10 times through an extruder using a filter with a pore size of 0.2 µm [20].

### 2.4. Fluorescence Spectroscopy Analysis

#### 2.4.1. Lipid Mixing Assay

Labeling with R18 was performed according to the following procedure. A mixture of LUV with a total lipid concentration of 400 mM was added with R18 and was used to obtain a lipid/R18 ratio of 100:1 and incubated for 1 h at room temperature. R18-labeled vesicles were washed with a 20 mM citrate buffer at pH 4.5 and centrifuged two times at 25,000 rpm at 4 °C for 30 min using a HITACHI CS 120GXL ultracentrifuge.

To perform the assays, R18-labeled vesicles and unlabeled vesicles were added to a quartz cuvette in a 1:1 ratio, with the total lipid concentration being 50 mM. Subsequently, each peptide was added separately as appropriate separately at 4 μM and then assays were performed in the presence of both peptides. While, to perform the negative control, assays were performed in the absence of both peptides, and for the positive control, triton X-100 was added at a final concentration of 0.5% *v*/*v* [10]. All steady-state fluorescence measurements were performed using a K2 spectrofluorimeter (ISS, Champaign, IL, USA). The fluorescence intensity of R18 was measured using an excitation wavelength of 560 nm and an emission wavelength of 590 nm. The fluorescence intensity was measured in each assay at 20 °C for 20 min and the % lipid mixing was obtained using Equation (1) [21]:

(1) $$\%\;lipid\;mixing=\frac{F-F_{0}}{F_{\infty }-F_{0}}\times 100$$

where *F* is the fluorescence intensity over time, *F*_0_ is the initial fluorescence, and *F_∞_* is the maximum fluorescence (100%), obtained by performing the assay in the presence of triton X-100.

#### 2.4.2. Determination of Binding Affinity Using Fluorescence Anisotropy

The anisotropy of peptide 303 was measured using tryptophan as an intrinsic probe to determine the dissociation constant (*Kd*) between the peptide and lipid vesicles. The measurements were performed using a 300 nm diode as the light source, and a 350 nm cut-off filter was used to record the fluorescence emission. For the measurement, we started by recording the fluorescence anisotropy of peptide 303 of 20 μM in a 20 μM citrate buffer at pH 4.5. Then, 5 μL of a LUV solution with a total lipid concentration of 800 μM was added to the previous solution and the fluorescence anisotropy was measured again; this process was performed until a final volume of added LUV of 100 μL was reached. *Kd* values were obtained from three independent assays by linear regression of 1/*r* (inverse anisotropy) versus 1/[*L*] (inverse lipid concentration) plots, as described by Pallicer et al [22]. The slope and intercept of the linear fit were used to calculate the dissociation constant.

(2) $$\frac{1}{r}=\frac{Kd}{r_{B}}\times \frac{1}{\left[L\right]}+\frac{1}{r_{B}}$$

#### 2.4.3. Generalized Polarization of Laurdan (GP)

A solution with 0.4 mM LUV prepared as above was incubated with 1 µM Laurdan for 30 min at 37 °C. To perform the Laurdan GP assays, a solution with LUV containing a lipid concentration of 100 µM was incubated for 2 min at 20 °C before each measurement was performed with peptide 303 at the concentrations of 0, 0.5, 1, 2, 3, and 4 μM. Measurements were performed using an excitation wavelength for Laurdan of 370 nm and recording emission at 440 nm and 490 nm. The GP was calculated using the following expression obtained from Parasassi et al [23]:

(3) $$GP=\frac{\left(I_{440}-I_{490}\right)}{\left(I_{440}+I_{490}\right)}$$

#### 2.4.4. Steady-State Fluorescence Anisotropy of DPH Probes

LUVs at a total lipid concentration of 0.4 mM were incubated with 0.5 µM DPH or TMA-DPH for 30 min at 60 °C. For anisotropy measurements, LUVs were diluted to a final lipid concentration of 100 µM, and peptide 303 was added to reach final concentrations of 0, 0.5, 1, 2, 3, and 4 μM. Each peptide–LUV mixture was incubated for 2 min at 20 °C prior to measurement. Steady-state fluorescence anisotropy was recorded using an excitation wavelength of 370 nm, and the emission was collected using 399 nm and 420 nm long-pass filters. Anisotropy values were calculated using Vinci software (ISS, Champaign, IL, USA) [24].

#### 2.4.5. Fluorescence Resonance Energy Transfer (FRET)

FRET assays were performed using an excitation wavelength of 474 nm, and fluorescence emission spectra were recorded from 500 to 700 nm using a spectrofluorimeter. The fluorescence spectrum of the donor peptide (ISAV-FP labeled with fluorescein) was first recorded at a concentration of 4 μM. Subsequently, increasing concentrations of the acceptor peptide (peptide 303 labeled with rhodamine B) were added (0.5, 1, 2, 2, 3, and 4 μM), and fluorescence spectra were recorded after each addition. The same procedure was repeated in the presence of LUVs at a total lipid concentration of 100 μM.

## 3. Results

To investigate the potential of peptide 303 as a modulator of viral membrane fusion, a series of fluorescence-based assays were conducted to evaluate its effects on lipid mixing, membrane organization, and direct interactions with the ISAV fusion peptide (ISAV-FP1). These experiments aimed to characterize the biophysical behavior of peptide 303 in model membrane systems and to determine if it could interfere with the fusogenic activity of ISAV-FP1. The results revealed distinct effects of peptide 303 on membrane fusion kinetics, the lipid bilayer order, and peptide–peptide interactions, providing mechanistic insights into its potential inhibitory role in viral membrane fusion.

### 3.1. Fluorescence Spectroscopy

#### 3.1.1. Lipid Mixing Assay

**Figure 2:**
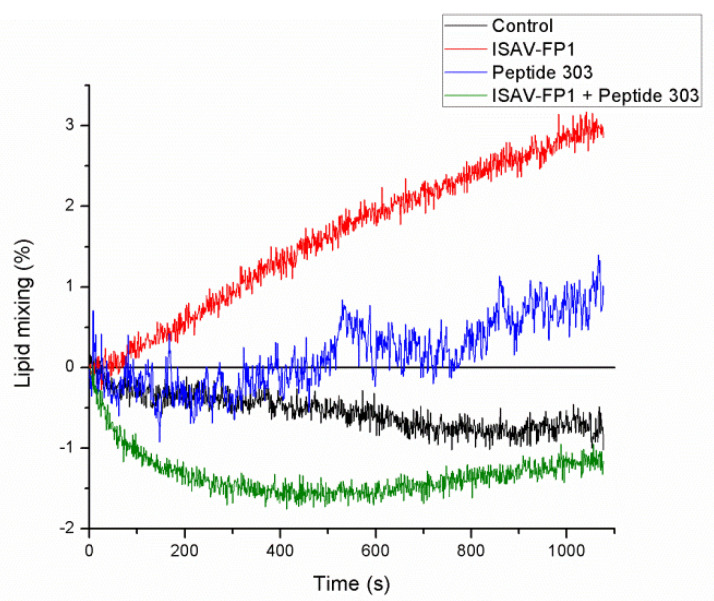
Kinetics of LUV lipid mixing mediated by the ISAV fusion peptide (ISAV-FP1, red line), peptide 303 (blue line), and the combination of both peptides (green line). The negative control (black line) is the peptide-free LUV fusion assay. Lipid mixing was expressed as a percentage relative to total fluorescence obtained upon the addition of 0.5% *v*/*v* triton X-100. Each curve represents the mean of three independent experiments.

To evaluate the membrane fusion capacity of the peptides, a lipid mixing assay was performed (Figure 2). In the presence of the ISAV-FP1 peptide, a time-dependent increase in membrane fusion was observed, reaching a maximum fusion level of approximately 3.17%. In contrast, peptide 303 induced a significantly lower degree of lipid mixing, with a maximum fusion of 1.40% and a moderate increase over time compared to ISAV-FP1. When both peptides were used in combination, ISAV-FP1 was added first, immediately followed by peptide 303; the lipid mixing values were negative and below those of the control condition, suggesting that peptide 303 may interfere with or inhibit the fusogenic activity of ISAV-FP1 under these conditions.

#### 3.1.2. Steady-State Fluorescence Analysis of Peptide 303 Interaction with LUVs and Fusion Peptides

To evaluate the interaction of peptide 303 with lipid membranes and with a viral fusion peptide, three groups of fluorescence-based assays were performed.

**Figure 3:**
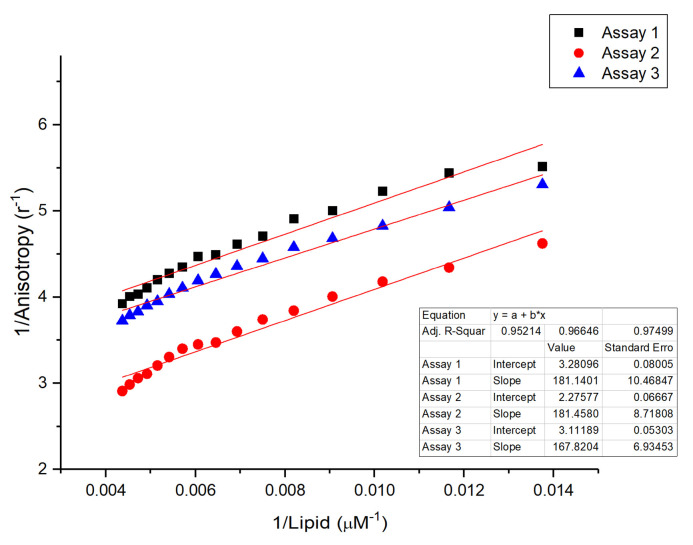
Anisotropy assays of peptide 303 to determine its dissociation constant (*Kd*) with large unilamellar vesicles (LUVs) composed of DOPC/DPPC/cholesterol in a 2:2:1 molar ratio. The assays were performed in triplicate, measuring the steady-state fluorescence anisotropy of peptide 303 after successive additions of defined amounts of LUVs. Experiments were conducted in a 20 mM citrate buffer at pH 4.5 and 20 °C.

First and as shown in Figure 3, the binding affinity of peptide 303 for lipid vesicles was assessed by determining the dissociation constant (*Kd*) using fluorescence anisotropy. A *Kd* value of 62.96 ± 14.54 μM was obtained, indicating a moderate affinity of peptide 303 for the lipid phase of LUVs.

**Figure 4:**
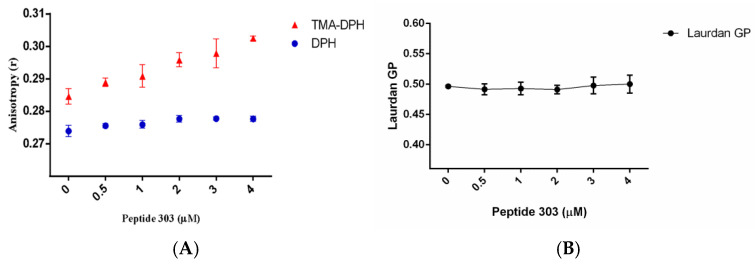
Laurdan generalized polarization (GP) and steady-state fluorescence anisotropy of DPH and TMA-DPH in LUVs (DPPC–DOPC–CHO, 2:2:1 molar ratio), in the absence and presence of peptide 303. (**A**) Fluorescence anisotropy measurements of DPH (hydrophobic core) and TMA-DPH (lipid–water interface) reveal peptide-induced changes in the membrane order at different depths of the bilayer. (**B**) Laurdan GP values reflect membrane packing and hydration near the glycerol backbone region. Data points correspond to the mean values from six independent experiments.

Second, as shown in Figure 4, to assess the effect of peptide 303 on lipid membrane organization and fluidity, membrane-incorporated fluorescent probes were employed. Fluorescent probes based on DPH have been extensively used to detect changes in lipid phase fluidity in membrane models such as LUVs. DPH inserts into the hydrophobic core of the lipid bilayer, aligning between the acyl chains of phospholipids. In contrast, TMA-DPH, due to the presence of its charged trimethylammonium group, localizes closer to the glycerol backbone of the lipids, sensing the outer region of the hydrophobic layer. The combined use of these probes allows the assessment of the lipid packing order at different depths of the bilayer, providing a spatially resolved view of membrane organization. TMA-DPH anisotropy assays (Figure 4A), which reflect the membrane order at the level of the outer region of the hydrophobic lipid phase, showed a significant increase in anisotropy values at all used concentrations of peptide 303 compared to the control (no peptide). This trend suggests that peptide 303 increases lipid packing or the order in the outer region of the bilayer. In contrast, DPH anisotropy measurements—reporting on the hydrophobic core of the membrane—showed no statistically significant changes across peptide concentrations, indicating that peptide 303 does not significantly affect the internal membrane order. Complementarily, Laurdan fluorescence was used to monitor changes in membrane hydration and fluidity at the lipid–water interface (see Figure 4B). The generalized polarization (GP) parameter did not vary significantly with increasing concentrations of peptide 303, suggesting that this peptide does not induce measurable changes in membrane hydration or global phase properties detectable by Laurdan.

**Figure 5:**
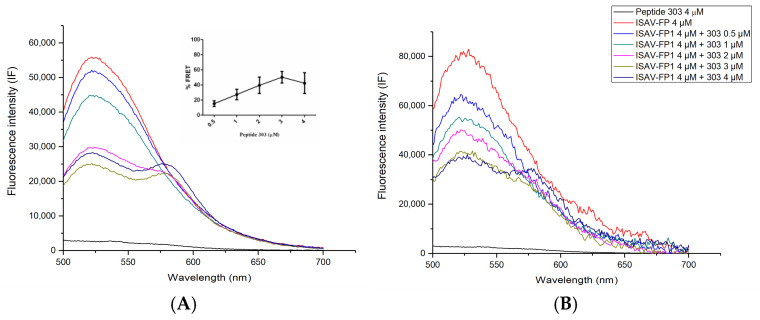
Förster Resonance Energy Transfer (FRET) assays between rhodamine B-labeled peptide 303 (acceptor) and fluorescein-labeled ISAV-FP1 (donor), performed to evaluate direct peptide–peptide interactions. Each condition was measured in triplicate. (**A**) FRET signal obtained in a solution (absence of LUVs). (**B**) FRET signal in the presence of large unilamellar vesicles (LUVs). Samples were excited at 474 nm, and emission spectra were recorded from 500 to 700 nm at 20 °C. As a control, the fluorescence spectrum of rhodamine-labeled peptide 303 (acceptor) was recorded in the absence of the donor (black line). Each curve represents the mean of three independent experiments.

Finally, to assess the potential for a direct interaction between peptide 303 and the ISAV fusion peptide, Förster Resonance Energy Transfer (FRET) assays were performed using fluorescein-labeled ISAV-FP1 as the donor and rhodamine B-labeled peptide 303 as the acceptor (Figure 5). As a control, the fluorescence spectrum of rhodamine-labeled peptide 303 (acceptor) was recorded in the absence of the donor, upon excitation at 474 nm, corresponding to the donor’s excitation wavelength. This technique enables the detection of molecular proximity at the nanometer scale, indicative of a physical interaction between the two peptides. A concentration-dependent increase in the percentage decrease in donor fluorescence intensity (%FRET) was observed when the donor concentration was held constant and increasing concentrations of the acceptor peptide were added, supporting the possible existence of a direct interaction between ISAV-FP1 and peptide 303 (Figure 5A; see inset). When the same experiment was carried out in the presence of LUVs, a similar trend was observed; however, the extent of energy transfer was reduced compared to the assays conducted in a solution. This suggests that membrane association may partially restrict or modulate the interaction between the peptides, possibly by altering their orientation or availability for complex formation (Figure 5B).

As shown in Figure 5, donor fluorescence decreases in the presence of the acceptor, which could suggest quenching due to peptide interactions or conformational changes. However, the simultaneous increase in acceptor emission at the rhodamine wavelength, along with a negligible signal from direct acceptor excitation controls, strongly supports energy transfer as the primary mechanism.

## 4. Discussion

Several studies have shown that peptide-based inhibitors of membrane fusion can modulate viral entry by interfering with the conformational rearrangements required for fusion protein function. For instance, peptides derived from viral fusion machinery, such as the HIV-1 gp41 six-helix bundle and the SARS-CoV-2 spike protein, have demonstrated potent inhibitory activity by preventing the formation of fusion-active conformations [25]. Similarly, coronin 1-derived peptides containing tryptophan-aspartic acid repeats were shown to disrupt content mixing by altering membrane organization and dynamics [26,27]. In our study, peptide 303 appears to act through a comparable mechanism by modifying the membrane order and interacting directly with the ISAV fusion peptide, ISAV-FP1.

Our membrane fusion assays demonstrated that peptide 303 inhibits ISAV-FP1-mediated lipid mixing. Although peptide 303 alone induced a modest increase in fusion compared to ISAV-FP1, the effect was considerably weaker. Importantly, when both peptides were present, no fusion was detected, suggesting a functional antagonism. This inhibitory effect could result from peptide 303 stabilizing the membrane order, as well as from a direct interaction with ISAV-FP1 that obstructs its fusogenic activity. The negative fusion values observed in this condition likely reflect a suppression of baseline lipid mixing, consistent with a membrane-stabilizing effect exerted by peptide 303.

The measured dissociation constant (*Kd* = 62.96 ± 14.54 µM) for peptide 303’s binding to lipid vesicles is similar to that reported for ISAV-FP1 (68.33 ± 3.73 µM) by Tarnok et al. [13], indicating comparable membrane affinity. This suggests that peptide 303 can independently associate with lipid bilayers—a prerequisite for modulating the membrane structure and interfering with fusion. This finding parallels reports on other viral fusion inhibitors that must integrate into the membrane to exert their effects.

To investigate how peptide 303 influences membrane organization, we performed steady-state fluorescence spectroscopy using membrane-sensitive probes. Laurdan GP assays, which report on membrane hydration and lipid packing near the glycerol backbone, showed no significant changes, suggesting that peptide 303 does not perturb this region. In contrast, TMA-DPH anisotropy increased significantly in a concentration-dependent manner, indicating that peptide 303 enhances the lipid order at the membrane hydrophobic–hydrophilic region. The absence of significant changes in DPH anisotropy, which reflects deeper hydrophobic core dynamics, suggests that this ordering effect is restricted to the outermost region of the lipid bilayer’s hydrophobic domain. These findings align with those of Pandia et al. [24], who showed that peptide inhibitors can selectively alter membrane dynamics at defined bilayer depths. Overall, our results indicate that peptide 303 does not affect the order or hydration of the polar headgroup region of the lipid bilayer, as evidenced by the absence of significant changes in Laurdan generalized polarization (GP) values. Similarly, no alterations were detected in the deeper hydrophobic core, as shown by the unchanged anisotropy of DPH. In contrast, a significant increase in lipid packing was observed in the upper region of the hydrophobic phase, as reported by TMA-DPH anisotropy. These findings suggest that peptide 303 preferentially inserts into the outer segment of the hydrophobic region, likely interacting with the glycerol backbone and proximal acyl chain area without perturbing the bilayer surface or core.

Interestingly, a previous study demonstrated that ISAV-FP1-mediated fusion is sensitive to the membrane order: increased cholesterol content led to higher lipid packing and reduced fusion efficiency, without affecting TMA-DPH anisotropy [13]. In contrast, peptide 303 not only inhibits fusion but also increases TMA-DPH anisotropy, highlighting its unique capacity to stabilize the membrane region critical for fusion initiation. This supports a model in which peptide 303 impairs the disordering of lipid tails necessary for hemifusion or pore formation.

Finally, FRET experiments confirmed a direct interaction between peptide 303 and ISAV-FP1. When both peptides were in a solution, a concentration-dependent increase in energy transfer was observed, indicating spatial proximity and likely complex formation. This interaction likely contributes to the inhibition of fusion by sequestering ISAV-FP1 or altering its functional conformation. Notably, the FRET signal persisted in the presence of lipid vesicles, albeit at reduced intensity. This suggests that while the peptides can still interact in a membrane context, the bilayer may partially limit their association, possibly due to changes in spatial orientation or competition for membrane insertion.

These findings are consistent with previous reports of viral fusion inhibitors that function through direct binding to viral fusion proteins. For example, in the Epstein–Barr virus, a peptide derived from gp42 binds with high affinity to the gH/gL complex and potently inhibits membrane fusion at nanomolar concentrations [28]. Our results suggest a similar mode of action for peptide 303 in the ISAV system, combining both membrane remodeling and direct peptide–peptide interactions as complementary mechanisms of inhibition.

Overall, our results demonstrate that peptide 303 effectively inhibits vesicle fusion mediated by the ISAV-FP1 fusion peptide. Moreover, we provide evidence that peptide 303 interacts with the lipid phase of LUVs. However, its interaction with the membrane could potentially disrupt the function of cellular membrane proteins, raising the possibility of cytotoxic effects. Therefore, future studies should address the cytotoxicity of peptide 303 to assess its safety profile.

In this context, peptide 303 emerges as a multifunctional inhibitor of ISAV-mediated membrane fusion, capable of both altering membrane biophysical properties and directly engaging the viral fusion peptide. This dual mechanism reflects broader strategies used by other antiviral fusion-inhibitory peptides and supports the potential of peptide 303 or its derivatives as candidates for the development of novel antiviral agents targeting orthomyxoviruses and related enveloped viruses.

## 5. Conclusions

In summary, the present study provides evidence that peptide 303 modulates the membrane interfacial lipid bilayer order and interferes with the fusogenic activity of the ISAV fusion peptide (ISAV-FP1) through two complementary mechanisms: altering the lipid bilayer order and directly interacting with the fusion peptide. The increase in anisotropy observed with the TMA-DPH probe, but not with DPH, indicates that peptide 303 selectively increases the lipid packing order in the hydrophobic–hydrophilic interface region of the lipid bilayer without significantly affecting the hydrophobic core. This localized ordering of the membrane could hinder the dynamic lipid rearrangements necessary for membrane fusion, a process that typically requires transient membrane destabilization and increased fluidity.

Moreover, peptide 303 showed a measurable affinity for lipid membranes, with a dissociation constant comparable to that of ISAV-FP1, supporting its capacity to insert independently into the bilayer. This insertion, in combination with the membrane-ordering effect, provides a biophysical basis for its ability to interfere with the fusion process.

Crucially, FRET experiments revealed a direct, concentration-dependent interaction between peptide 303 and ISAV-FP1, both in a solution and in the presence of membranes. Although the efficiency of energy transfer was reduced in the lipid context, the persistence of FRET signals suggests that peptide 303 can interact with ISAV-FP1 in a membrane-associated state. This interaction likely contributes to the observed inhibition of fusion, not only by altering membrane physical properties but also by acting as a competitive or allosteric inhibitor that prevents ISAV-FP1 from adopting its fusogenic conformation or assembling into functional complexes.

Taken together, these findings suggest that peptide 303 exerts its inhibitory effect through a dual mechanism—the biophysical modulation of the lipid environment and direct molecular interference with the fusion peptide. This duality reinforces the importance of both membrane composition and peptide–peptide interactions in regulating viral fusion processes. From a broader perspective, these results underscore the potential of membrane-active peptides as modulators of viral entry, offering insights that could inform the rational design of fusion inhibitors targeting similar envelope viruses.

## Data Availability

The raw data supporting the conclusions of this article will be made available by the authors upon request.

## Abbreviations

The following abbreviations are used in this manuscript:

ISAV	Infectious Salmon Anemia Virus
ISAV-FP1	ISAV fusion peptide
ISA	Infectious Salmon Anemia
LUVs	large unilamellar vesicles
DPPC	dipalmitoylphosphatidylcholine
DOPC	1,2-dioleoyl-sn-glycero-3-phosphocholine
HE	hemagglutinin esterase
DPH	1,6-diphenyl-1,3,5-hexatriene
TMA-DPH	1-(4-trimethylammonium)-6-phenyl-1,3,5-hexatriene
Laurdan	6-lauroyl-2-dimethylaminonaphthalene
R18	octadecyl rhodamine B chloride
TX-100	triton X-100
SPPS	solid-phase peptide synthesis
Fmoc	9-fluorenylmethoxycarbonyl
Kd	dissociation constant
GP	generalized polarization
FRET	Förster Resonance Energy Transfer
